# Impact of Quarantine During COVID-19 Pandemic on the Quality of Life of Patients with Allergic Conjunctivitis

**DOI:** 10.7759/cureus.12240

**Published:** 2020-12-23

**Authors:** Walaa Al-Dairi, Ali A Al Saeed, Omar M AL Sowayigh

**Affiliations:** 1 Surgery, King Faisal University, Al Ahsa, SAU; 2 College of Medicine, King Faisal University, Al Ahsa, SAU

**Keywords:** allergic conjunctivitis, covid-19, quarantine, ocular allergy, preventive ophthalmology

## Abstract

Introduction

The conjunctiva is one of the most frequently damaged targets for inflammatory responses induced by allergic immunological hypersensitivity reactions. Allergic conjunctivitis (AC) typically manifests as a spectrum of potential severity, ranging from mild symptoms to severe troublesome symptoms that might interfere significantly with daily activities and overall quality of life.

Aim

This study aimed to evaluate impact of the quarantine during the COVID-19 pandemic on the quality of life of allergic conjunctivitis patients.

Methodology

This is a cross-sectional study conducted among confirmed patients with allergic conjunctivitis in Saudi Arabia from the period of July 2020 to September 2020. An electronic validated structured-questionnaire explored the participants' demography, symptoms, known allergen and risk factors, and patient psychological and functional domains of life using Eye Allergy Patient Impact Questionnaire (EAPIQ). Data were gathered in MS Excel and all statistical analyses were performed using SPSS version 21.

Results

The most common symptoms of AC was itching (79.9%) and redness of the eye (38.8%), while the most common causes was dust (46.9%) and pollens (46.9%). With regards to the assessment of EAPIQ, the mean score was higher in the impact of eye allergy symptoms domain (mean 12.6 ± 4.84 SD.), while it was lower in the impact of AC on the psychological and emotional domain (mean 10.7 ± 5.62 SD.). Statistical analysis revealed that previous history of AC and a visit to healthcare provider were the significant factors associated with the increased risk of eye allergy symptoms, troubled daily activities and bothered psychological and emotional life.

Conclusion

This study demonstrated that a patients' eye allergy symptoms affect many aspects of patients' daily activities. In addition to affecting functionality, these symptoms also affect patients' emotional state. Although the impact of daily activities and emotions due to eye allergy were minimal, the impact of eye allergy symptoms during quarantine period was still found to be moderate. We found the severity of AC & its impact over a patients’ quality of life was moderate which suggests there was no major effect found on AC severity during the COVID-19 pandemic quarantine period. Further studies with detailed analysis of triggering factors might lead to a better understanding of the disease and its relation to the patients’ activities & lifestyle which can directly affect the AC management & quality of life.

## Introduction

The conjunctiva notably is the most immunologically sensitive tissue of the external eye and is frequently undergoing lymphoid hyperplasia in response to a limitless variety of local and systemic stimulants [1]. Allergic conjunctivitis (AC) is a localized allergic condition that is often associated with rhinitis and asthma; however, ocular allergy can be the only troublesome allergic symptom [2]. Allergic conjunctivitis typically manifests as a spectrum of potential severity, ranging from mild symptoms like red, itchy, burning, swollen, or dry eyes in differing degrees of severity and duration. While some patients might barely be affected for a few weeks, others may intensively suffer from troublesome symptoms continuously throughout the year that might interfere significantly with their daily activities and overall quality of life [3,4,5].

Till date, the accepted etiological theory of AC is believed to be due to multifactorial and complex interactions of numerous factors that take part in the pathogenesis of AC, including genetics, animal dander, early childhood exposure and air pollution in urban areas [6]. Air pollution is well-recognized for it's negative impact on human health, especially allergic diseases including AC. Numerous studies have invariably found that air pollution aggravates asthma and can even trigger its onset [7]. However, very few published studies have adequately evaluated the direct relationship between air pollution and ocular allergic diseases [8].

Coronavirus disease 2019 (COVID-19) is a new, rapidly emerging, and highly contagious disease. On March 11, 2020, the World Health Organization declared COVID-19 as a pandemic disease [9]. As a preventive measure, Saudi Arabia and most of the world’s countries imposed compulsory lockdowns to reduce the spread of the virus. The effect of restricted human activities due to the COVID-19 pandemic resulted in a significant drop in air pollution and air particles [10]. We hypothesize that quarantine during the pandemic of COVID-19 improved the quality of life of patients with AC, since quarantine and human activity restrictions led to a marked drop in air particles and air pollution and reduction in the exposure to possible outdoor stimulants [10], which might have led to a decrease in the frequency of episodes AC or at least it’s severity. Therefore, the ultimate aim of the present study is to evaluate the impact of quarantine on the frequency and episodes of symptomatic AC alongside with the psychological impact of AC.

## Materials and methods

This is a descriptive cross-sectional study conducted among patients confirmed with AC in Saudi Arabia from the period of July 2020 to September 2020. The selection was based on an allergic conjunctivitis symptoms questionnaire. An electronic validated structured-questionnaire explored the participants' demographical characteristics, symptoms, known allergen and risk factors, and impact on patient psychological and functional domains of life using Eye Allergy Patient Impact Questionnaire (EAPIQ). The questionnaire was pretested in a pilot study in Saudi Arabia before using it in this study. Data were gathered in MS Excel and all statistical analyses were performed using SPSS version 21.

Scoring criteria

The questionnaire was derived from Eye Allergy Patient Impact Questionnaire (EAPIQ) [11]. It is composed of three domains: impact of eye allergy symptoms, impact of AC on daily activities and impact of AC on psychological and emotional life. Each domain contains 5 statements and has a 5-point Likert scale category ranging from “not troubled” coded as 1 to “extremely troubled” coded as 5 or “none of the time” coded as 1 to “all the time” coded as 5. The total score for each domain was calculated by adding the scores of all questions each, separately. The scores generated have a minimum of 5 points and maximum of 25 points. The overall EAPIQ score was calculated by adding 15 questions of the three domains divided by 3. This indicates that the higher the score, the higher the impact of eye allergy into their everyday life [11].

Inclusion criteria

**Figure 1:**
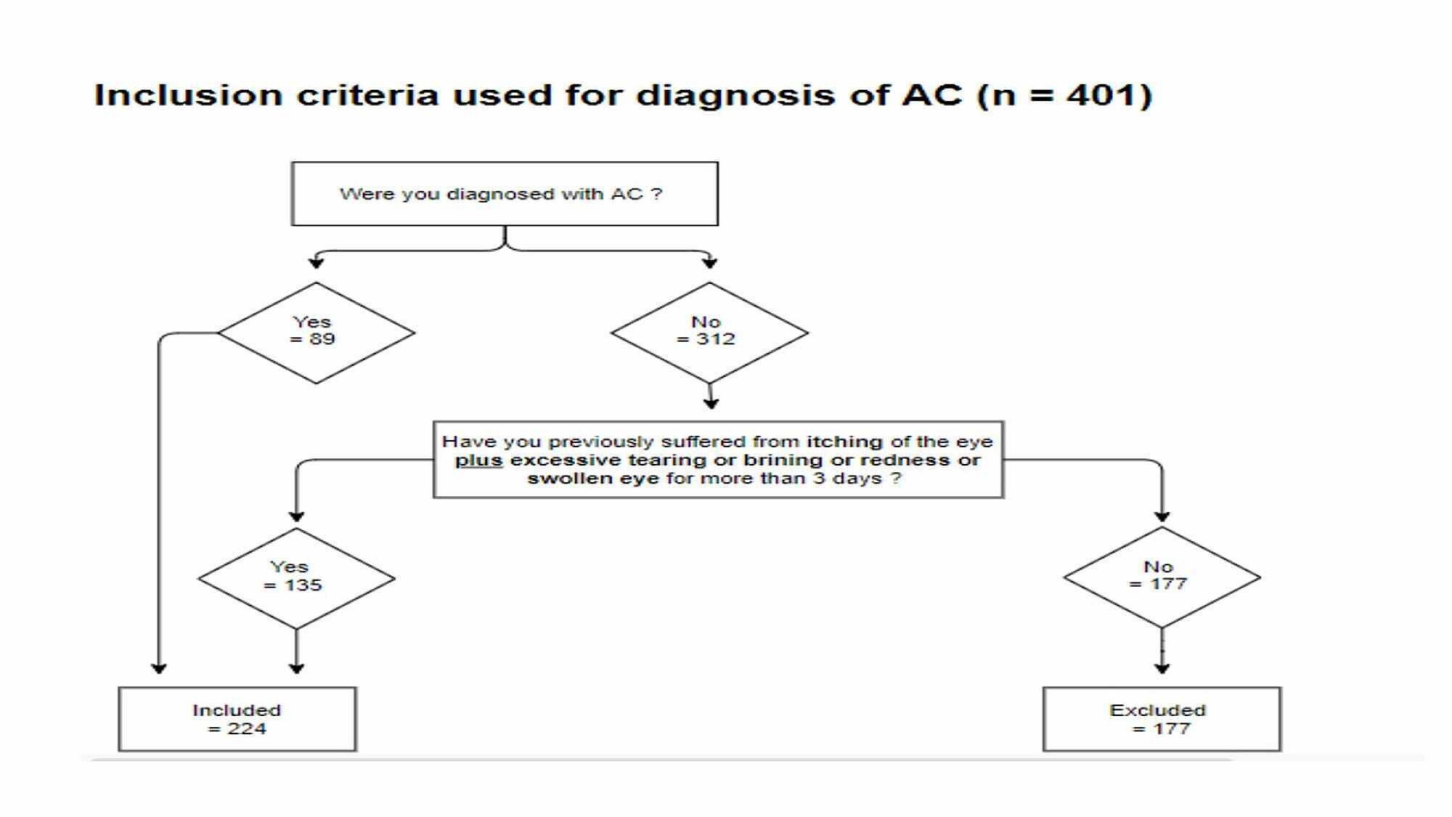
Inclusion Criteria for diagnosis of AC AC : Allergic conjunctivitis

Due to the limited access into ophthalmology patients during the COVID-19 pandemic, we used the questionnaire with specific symptoms analysis inclusion criteria to diagnose AC. The diagnostic criteria was discussed and approved by three consultants of ophthalmology. With an estimated population of 33.7 million and a prevalence of AC in Saudi Arabia estimated to be 70.5%, the sample size was estimated to be 384. In this study we have reached 401 patients [12]. As illustrated in Figure 1, we included all previously diagnosed patients with AC and patients who were diagnosed by a clinician. If not diagnosed, then participants having itching plus one or more of the following symptoms for more than 3 days: redness, tearing and puffiness/swollen eye were included in the study. Out of 401 participants only 224 met the criteria.

Statistical analysis

The data was shown as frequency, percentage, minimum, maximum, mean and standard deviation, whenever appropriate. The mean scores were compared between the subgroups using a Mann Whitney U test (non-parametric). Normality tests were conducted using Shapiro Wilk test. Correlation studies were conducted to determine the linear relationship between EAPIQ total score and its domains. P values <0.05 were considered statistically significant. All statistical analyses were performed using the Statistical Package for the Social Sciences (SPSS) software, for Windows (version 21; IBM, Armonk, New York).

## Results

We recruited 224 patients who were either following up with AC or fulfilled the AC diagnostic criteria. Table 1 presented the sociodemographic characteristics of the patients. The age range was from 17 to 65 years old (mean 30.8) with nearly 60% patients in the younger age group (<30 years old). Furthermore, more than a half were females (58%) and nearly all were Saudis (94.6%). With respect to the nature of their job, nearly all were working indoors (92%). The prevalence of patients with previous history of AC was 39.7%.

**Table 1:** Socio demographic characteristics of the patients (n=224)

Study data	N (%)
Age group (mean ± SD)	30.8 ± 11.2
<30 years	131 (58.5%)
≥30 years	93 (41.5%)
Gender	
Male	94 (42.0%)
Female	130 (58.0%)
Nationality	
Saudi	212 (94.6%)
Non-Saudi	12 (05.4%)
Job nature	
Indoor	206 (92.0%)
Outdoor	18 (08.0%)
Previous history of allergic conjunctivitis	
Yes	89 (39.7%)
No	135 (60.3%)

**Figure 2:**
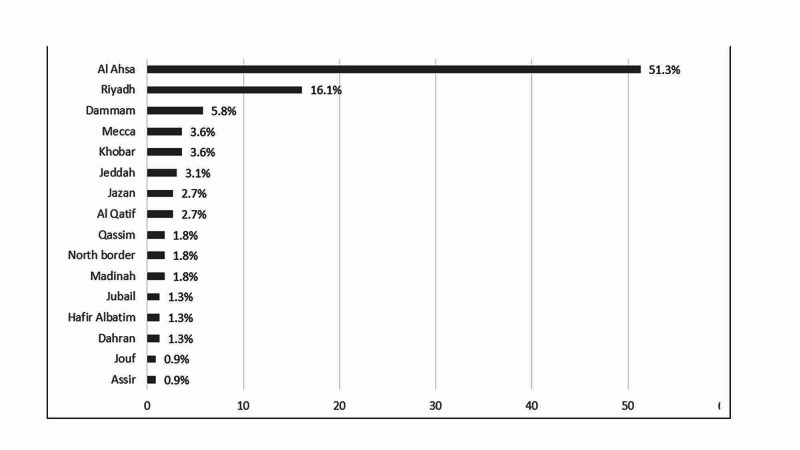
Distribution of residence city (n=224)

Figure 2 shows the distribution of residence city. It was revealed that the most commonly mentioned city was Al Ahsa (51.3%), followed by Riyadh [16.1%) and Dammam (5.8%) while Assir and Jouf were the least mentioned (each 0.9%, respectively).

**Figure 3:**
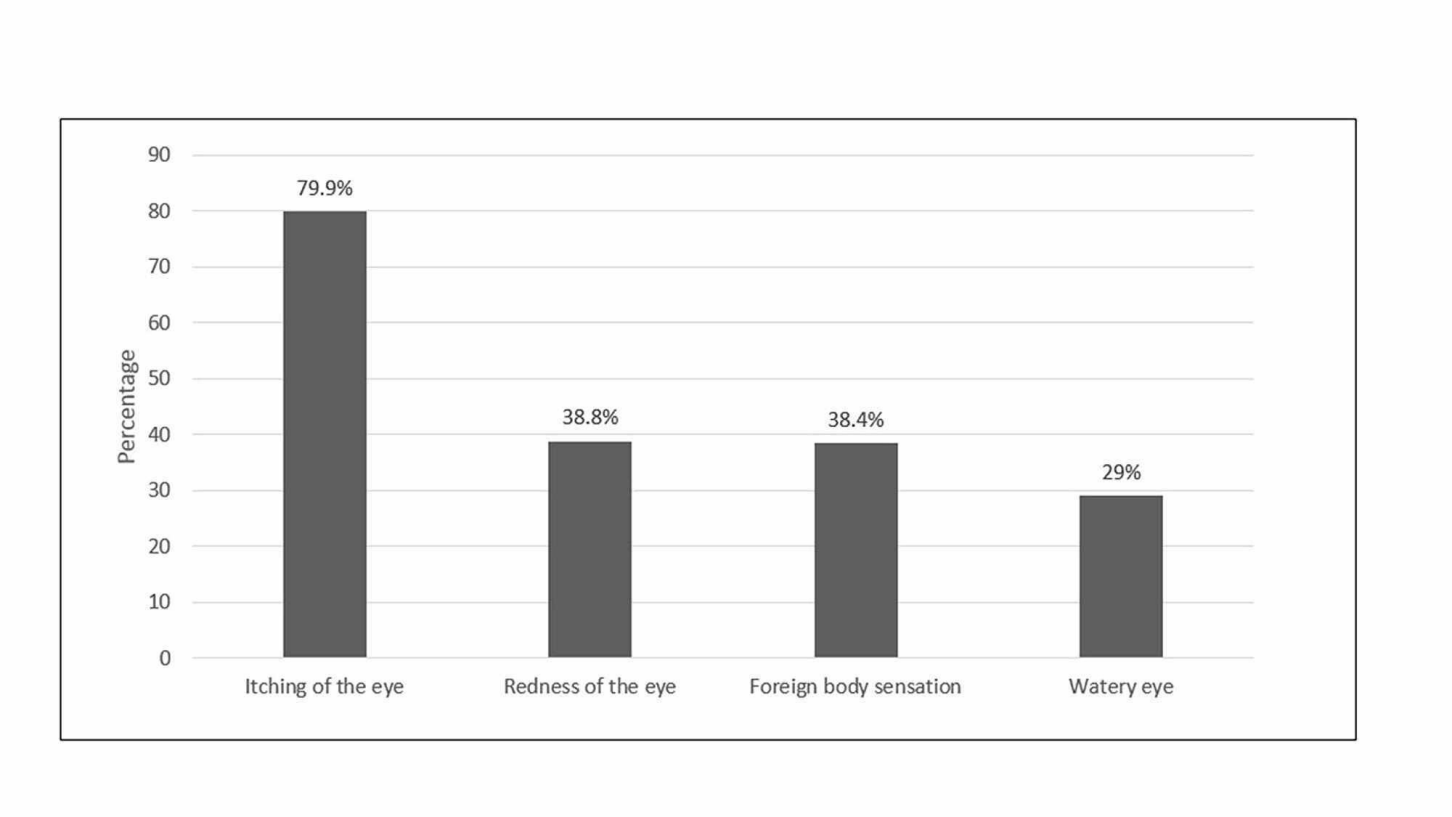
Symptoms of Allergic Conjunctivitis (n=224)

In figure 3, the most frequently mentioned symptoms of AC were itching of the eye (79.9%), followed by redness of the eye (38.8%) while watery eye was the least (29%).

Table 2 describes the characteristics of patients with symptoms of AC. It was found that the most common causes of AC were dust (46.9%), followed by pollen (14.3%) and contact lenses (12.1%), while the least of them was animal dander (7.6%). It was also observed that while 66.1% of the patients reported that AC recurrence had no specific time to occur over the year, 10.7% report that it occurred during summer and 8% reported it might occurred during winter. Furthermore, 32.6% were taking AC medication along with artificial teardrops. In addition, among 46 patients who visited a healthcare provider, the most frequently visited was optometrist/ophthalmologist (60.9%) and pharmacist/chemist (30.4%).

**Table 2:** Characteristics of patients with symptoms of Allergic Conjunctivitis (n=224)

Variables	N (%)
Causes of AC	
Dust	105 (46.9%)
Pollen	32 (14.3%)
Contact lenses	27 (12.1%)
Perfume	21 (09.4%)
Animal dander	17 (07.6%)
Others	01 (0.40%)
Season of AC recurrence	
No specific time of the year	148 (66.1%)
Summer	24 (10.7%)
Winter	18 (08.0%)
Summer and spring	09 (04.0%)
Spring	06 (02.7%)
Summer and fall	05 (02.2%)
Fall	03 (01.3%)
All over the year	03 (01.3%)
Winter and summer	03 (01.3%)
Taking medication	
Yes	73 (32.6%)
No	151 (67.4%)
Specific type of medication ^(n=73)^	
Topical medications	64 (87.7%)
Oral antihistamine	02 (02.7%)
Others	07 (09.6%)
Healthcare provider visits during quarantine ^(n=46)^	
General practitioner/Internist	02 (04.3%)
Optometrist/Ophthalmologist	28 (60.9%)
Allergist	02 (04.3%)
Pharmacist/Chemist	14 (30.4%)

Table 3 describes the assessment of EAPIQ. Based on the results, the impact of eye allergy symptoms has the highest score (mean 12.6 ± 4.84), followed by impact of AC on daily activities (mean 10.9 ± 4.99 SD), while the impact of AC on the psychological and emotional life (mean 10.7 ± 5.62) was the lowest. The overall mean EAPIQ score was 11.4 ± 4.27.

**Table 3:** Assessment of Eye Allergy Patient Impact Questionnaire (EAPIQ) (n=224) Legend: 5 – 12 (Mild symptoms); >12 – 18 (Moderate symptoms); >18 – 25 (severe symptoms).

Domain	Mean ± SD	Median (min – max)	Perceived rating
Eye allergy symptoms	12.6 ± 4.84	12 (5 – 25)	Moderate symptoms
Daily activities	10.9 ± 4.99	10 (5 – 25)	Mild symptoms
Psychological and emotional life	10.7 ± 5.62	09 (5 – 25)	Mild symptoms
Total Score	11.4 ± 4.27	10.7 (5 – 22.3)	Mild symptoms

Table 4 presented the correlation between EAPIQ score and its domain. It was revealed that the correlation between eye allergy symptoms among daily activities (r=0.615; p<0.001), psychological and emotional life (r=0.480; p<0.001) and total score (r=0.828; p<0.001) were positively & highly statistically significant. It was also observed that daily activities were positively highly correlated with psychological and emotional life (r=0.503; p<0.001) and total score (r=0.843; p<0.001) while the correlation between total score and psychological and emotional life were also positively correlated (r=0.816; p<0.001). In summary, this indicates that the increase of each domain is associated with the increase of the total EAPIQ score.

**Table 4:** Correlation (Pearson-r) between EAPIQ score and its domain (n=224) ** Correlation is significant at the 0.01 level (2-tailed).

SN	Domain of AC	I	II	III	IV
I	Eye allergy symptoms	1			
II	Daily activities	0.615 **	1		
III	Psychological and emotional life	0.480 **	0.503 **	1	
IV	Total Score	0.828 **	0.843 **	0.816 **	1

Table 5demonstrates the relationship in between EAPIQ domain and sociodemographic characteristics of participants. When measuring the association between the EAPIQ domains mean score and the sociodemographic characteristics of the patients, it was found that those in the younger age group (< 30 years) were significantly better and more likely to manage with their daily activities (T=-3.043; p=0.005). It was also observed that male participants showed a significantly better psychological and emotional health than females (T=-3.103; p=0.002). Similarly, those working outdoors were better at dealing with daily activities (T=0.375; p=0.034) and psychological and emotional health (T=0.774; p=0.039). On the other hand, non-Saudi patients were observed to have significantly affected psychological and emotional health (T=-2.542; p=0.024) than Saudis. Similarly, those who were living outside Al Ahsa were found to be more associated with eye allergy symptoms (T=-2.785; p=0.006). Likewise, those with previous history of AC exhibited a higher risk of eye allergy symptoms (T=2.278; p=0.032), troubled daily activities (T=2.797; p=0.019) and affected psychological and emotional health (T=2.464; p=0.005), while those who were taking medication were significantly associated with more eye allergy symptoms (T=4.064; p<0.001) and troubled daily activities (T=3.839; p<0.001). Additionally, those who visited a healthcare provider were likely to be associated with more eye allergy symptoms (T=4.571; p<0.001), troubled daily activities (T=2.910; p=0.010) and affected psychological and emotional health (T=3.521; p<0.001).

**Table 5:** Statistical Association between EAPIQ domains and the socio demographic characteristics of the patients [n=224) P-value has been calculated using Mann Whitney U test. ** Significant at p<0.05 level

Factor	Eye Allergy Symptoms Total Score [25) Mean ± SD	Daily Activities Total Score [25) Mean ± SD	Psychological and Emotional life Total Score [25) Mean ± SD
Age group [mean ± SD)			
<30 years	12.5 ± 4.91	10.1 ± 4.53	10.4 ± 5.63
≥30 years	12.6 ± 4.76	12.1 ± 5.39	11.2 ± 5.59
T-test; P-value	-0.129; 0.906	-3.043; 0.005 **	-1.121; 0.197
Gender			
Male	11.9 ± 4.69	10.3 ± 4.45	9.36 ± 4.97
Female	13.1 ± 4.88	11.4 ± 5.32	11.7 ± 5.87
T-test; P-value	-1.943; 0.057	-1.684; 0.184	-3.103; 0.002 **
Nationality			
Saudi	12.5 ± 4.88	10.8 ± 4.91	10.5 ± 5.47
Non-Saudi	14.2 ± 3.93	13.4 ± 5.99	14.7 ± 6.88
T-test; P-value	-1.166; 0.183	-1.786; 0.137	-2.542; 0.024 **
Residence city			
Inside Al Ahsa	11.7 ± 4.59	10.0 ± 4.12	9.84 ± 5.09
Outside Al Ahsa	13.5 ± 4.95	11.9 ± 5.64	11.6 ± 6.01
T-test; P-value	-2.785; 0.006 **	-2.863; 0.619	-2.384; 0.318
Job nature			
Indoor	12.7 ± 4.88	10.9 ± 4.97	10.8 ± 5.59
Outdoor	11.7 ± 4.29	10.5 ± 5.37	9.72 ± 5.87
T-test; P-value	0.788; 0.463	0.375; 0.034 **	0.774; 0.039 **
Previous history of AC			
Yes	13.5 ± 5.21	12.1 ± 5.53	11.8 ± 5.72
No	11.9 ± 4.49	10.2 ± 4.48	9.96 ± 5.44
T-test; P-value	2.278; 0.032 **	2.797; 0.019 **	2.464; 0.005 **
Taking medication			
Yes	14.4 ± 4.60	12.7 ± 5.19	11.8 ± 5.97
No	11.7 ± 4.71	10.1 ± 4.68	10.2 ± 5.38
T-test; P-value	4.064; <0.001 **	3.839; <0.001 **	1.954; 0.060
Visit to Healthcare provider			
Yes	15.5 ± 5.84	12.9 ± 5.68	13.3 ± 5.67
No	11.9 ± 4.31	10.5 ± 4.71	10.1 ± 5.43
T-test; P-value	4.571; <0.001 **	2.910; 0.010 **	3.521; <0.001 **

## Discussion

This study aimed to evaluate the impact of quarantine during the COVID-19 pandemic on the quality of life of allergic conjunctivitis patients using the EAPIQ questionnaire. Till date, there were no similar studies that discussed this issue. Thus, the findings of this study are important to understand the overall impact of quarantine period in the recurrence of AC. In this study, we discovered that the perceived impact of eye allergy symptoms was deemed moderate, and the overall means score of eye allergy was 12.6 (SD 4.84) out of 25 points (higher the score, higher impact to eye allergy symptoms). Consequently, the impact of daily activities and psychological health to eye allergy during quarantine period were minimal. Since there was pre-quarantine data documented in Saudi Arabia, we have compared our quarantine data to pre-quarantine data recently published in literature.

These results concur with the findings of Palmares et al. [3], a cross-sectional study that measured the clinical characteristics of AC and quality of life. According to their criteria, in a 10-point severity scale where 10 was the most severe, they found that 25.8% of the patients were classified their AC in the moderate level (4- 5), 45.6% were above 6 and only 5.1% considered their AC as mild. In India [13], Dayal et al. retrospectively investigated the trend of allergic rhinitis post the COVID-19 pandemic. They found out that there was a decrease in the trend of allergic rhinitis during the lockdown. They argued that the increased indoor activities were likely be the reason for the decreasing trend. This corroborated the study of Dror et al. [14] where a similar trend of decreasing allergic diseases were shown while using a face mask during the COVID-19 pandemic. They explained that a significant reduction in self-reported allergic rhinitis symptoms among nurses in wearing a face mask during the COVID-19 pandemic had been noted [14].

There were several factors that attributed to the increasing or decreasing impact of eye allergy symptoms. For example, living outside Al Ahsa, previous history of AC, taking allergy medications and visiting a healthcare provider, all linked to having a significant impact to allergy symptoms. On the other hand, those in the younger age group [< 30 years] and able to conduct outdoor activities during the quarantine period were less likely to have allergy symptoms, which might be explained by good medical control or no exacerbation at that time. On the other hand, those with previous history of AC, those who were taking medication and those who visited a healthcare provider were found to have impaired daily activities during the quarantine. Apparently, in our study, males were not greatly affected emotionally due to a flare up of an eye allergy. However, being non-Saudi, a previous history of AC and visits to healthcare provider has been strongly linked to disturbed emotional health. Conversely, Buchholz et al. reported that males were more often troubled by emotions due to allergy symptoms. [15].

It can be noted that the prevalence of patients with previous history of AC was 39.7%, which is consistent with the Palmares et al. study which reported a prevalence of 37.2%. Furthermore, the most frequent known symptoms of AC were itching of the eye which was found by Palmares et al. [3] as well as Buchholz et al [14]. Similarly, the most frequent causes of AC were dust followed by pollens and contact lens. These causes of AC are not in accordance with the study of Miyazaki et al. [16] where they indicated that cedar and cypress pollen represent the most common causes of AC. This difference reflects the variability of most common allergens according to geographical area. It also important to note that in this study, the majority of patients reported AC recurrences with no specific time or season during the year in Portugal [3].

The complains of AC happens mostly during spring or summer, while in Germany [15], the peak season for AC is during the month of April, May or June. Moreover, the prevalence of patients who were taking medication for the treatment of AC was 32.6% wherein topical medications were the most commonly used treatment. The prevalence of patients who were taking medication was higher in the study conducted in Portugal [3] and in Germany [15]. It is also worth mentioning that 20.5% of the patients visited a healthcare provider for the treatment of AC with a majority of them [60.5%] visiting an optometrist/ophthalmologist. This was higher than the study of Palmares et al. [3], with 19.4% of ophthalmologist visitations for the treatment of AC.

Limitation

A major limitation in this study is its cross-sectional nature where it lacks a patients’ follow up. Furthermore, the unknown level of indoor pollution and the fact that AC triggers might present at home limits the magnitude of real exposure. Thus, in future studies, some attempt should be made to evaluate the outdoor and indoor level of pollution including workplaces. This will provide a precise answer pointing out if being outdoors carries a higher risk in triggering allergic conjunctivitis episodes and increasing its severity or not.

## Conclusions

Based on previous studies, the prevalence of AC in Saudi Arabia is 70.5%, which is supported by the findings of this study. We evaluated the disease symptomatology and its severity. In addition to this, an evaluation to the disease impact on quality of life during quarantine for this specific population was performed. The impact on daily activities and emotions due to eye allergy were minimal and the impact of eye allergy symptoms during quarantine period was still found to be moderate. Previous history and a healthcare provider visit linked to a greater impact of eye allergy than the others. Further research is needed to validate the impact of quarantine period on AC. Further studies with a multi-center approach and detailed analysis of triggering factors might lead to a better understanding of the disease and its relation to the patients’ activities & lifestyle which can directly reflect on AC management & quality of life.
